# Efficacy of Laparoscopic Mini Gastric Bypass for Obesity and Type 2 Diabetes Mellitus: A Systematic Review and Meta-Analysis

**DOI:** 10.1155/2015/152852

**Published:** 2015-06-17

**Authors:** Yingjun Quan, Ao Huang, Min Ye, Ming Xu, Biao Zhuang, Peng Zhang, Bo Yu, Zhijun Min

**Affiliations:** ^1^Department of Gastrointestinal Surgery, Shanghai Pudong Hospital, Fudan University Pudong Medical Center, No. 2800, Gongwei Road, Shanghai 201399, China; ^2^Liver Cancer Institute, Zhongshan Hospital, Fudan University, No. 180, Fenglin Road, Shanghai 200032, China

## Abstract

*Background*. Controversies on the utility of laparoscopic mini gastric bypass (LMGB) in weight loss and type 2 diabetes mellitus (T2DM) control still exist. *Methods*. We conducted a comprehensive literature search of PubMed, EMBASE, and Cochrane Library. Review Manager was used to perform the meta-analysis and the weighted mean difference (WMD) and/or odds ratio with 95% confidence interval (95% CI) were used to evaluate the overall size effect. *Results*. The literature search identified 16 studies for systematic review and 15 articles for meta-analysis. Compared with LAGB, LSG, and LRYGB, LMGB showed significant weight loss [WMD, −6.58 (95% CI, −9.37, −3.79), *P* < 0.01 (LAGB); 2.86 (95% CI, 1.40, 5.83), *P* = 0.004 (LSG); 10.33 (95% CI, 4.30, 16.36), *P* < 0.01 (LRYGB)] and comparable/higher T2DM remission results [86.2% versus 55.6%, *P* = 0.06 (LAGB); 89.1% versus 76.3%, *P* = 0.004 (LAGB); 93.4% versus 77.6%, *P* = 0.006 (LAGB)]; LMGB also had shorter learning curve and less operation time than LRYGB [WMD, −35.2 (95% CI, −46.94, −23.46)]. *Conclusions*. LMGB appeared to be effective in weight loss and T2DM remission and noninferior to other bariatric surgeries. However, clinical utility of LMGB needs to be further validated by future prospective randomized controlled trials.

## 1. Introduction

Chronic diseases as the predominant death cause are well established, and obesity, being one of the factors strongly contributive to chronic diseases, has being consistently threatening the global health [1]. Obesity leads to multiple comorbidities including hypertension, hyperlipidemia, and hyperglycemia, whereas weight loss is associated with reduced metabolic and cardiovascular risks [2]. Specially, for obese people in prediabetes condition, weight control could lower the risk or delay the onset of type 2 diabetes mellitus (T2DM), with strict calorie restriction even reversing the progression of T2DM in established patients [3].

Bariatric surgery has long been introduced for weight control in conservative treatment failed individuals [4, 5] and was widely accepted in the past decades. Compared with nonsurgical strategies, bariatric surgery proves more effective for moderately to severely obese people to lose weight [6]. Besides, bariatric surgery was demonstrated to induce significant and long-term remission of T2DM [7, 8] and improvement of metabolic/cardiovascular risk factors in severely obese patients [9]. The short-term (decreased caloric intake) and long-term results (decreased fat mass and body weight) of bariatric surgery complementarily lead to improvement in glucose metabolism, insulin resistance, change in adipocytokines release [10], and quality of life [11]. Currently, bariatric surgery is well accepted as a feasible therapeutic option for T2DM management in patients who are inadequately controlled by healthy lifestyle and medical treatment [12].

Up to date, several bariatric surgeries exist [13]. Laparoscopic adjustable gastric banding (LAGB), laparoscopic sleeve gastrectomy (LSG), and laparoscopic Roux-en-Y gastric bypass (LRYGB) are the three most commonly used bariatric surgeries and LRYGB, accompanied with more rapid and more substantial weight loss than “restrictive” procedure (LAGB) and less risk of failure or complication than the “malabsorptive” procedure (LSG), is generally considered as the “gold standard” procedure [14]. Laparoscopic mini gastric bypass (LMGB) is the simplified procedure of LRYGB [15]. Upon its appearance, the prevalence of LMGB has been slow and lots of controversies arose. Complications including marginal ulcers, chronic alkaline reflux, Barrett esophagus, anastomosis leakage, and stenosis and requiring revisional surgery made it less popular; however, LMGB also has some advantages, such as one less anastomosis, shorter operative time (OT), lower risk of anastomotic leakage and internal herniation, shorter learning curve, and the ease of reversibility [16]. Unfortunately, there are no large scale multicenter randomized controlled trials to evaluate the clinical value of LMGB in comparison with other techniques and the indications and outcomes of LMGB in obese patients are still inconclusive. To this end, we conducted this systematic review and meta-analysis to evaluate and compare the efficacy, advantages, and complications of LMGB with those of LAGB, LSG, and LRYGB, trying to find some evidences to support the use of LMGB in treating obesity and T2DM.

## 2. Materials and Methods

### 2.1. Literature Searching and Study Selection

We conducted the literature searching by retrieving the electronic database of PubMed, EMBASE, and the Cochrane Library from inception until December 2014. The terms used were “mini gastric bypass,” “single anastomosis gastric bypass,” “omega loop gastric bypass,” “loop gastric bypass,” “MGB,” or “LMGB.” In addition, references of included literatures were retrieved manually for further evaluation. Two authors (Quan and Huang) independently performed the literature searching and the results were cross-checked to reach a consensus.

Studies were selected if they reported the outcomes of LMGB, compared LMGB with one or more bariatric procedures, and presented parameters of body weight index (BMI), waist circumference (WC), remission rate of T2DM, percentage of excess weight loss (%EWL), and other obesity or diabetes related factors as surgical effects. Conference abstracts were omitted since no detailed information about patient characteristics, interventions, or results could be achieved. If two or more studies from the same center or author were retrieved, the latest or the one with the largest sample size was selected. Studies were chosen for systematic review if they only reported outcomes of LMGB and for meta-analysis if comparisons between LMGB and LAGB, LSG, or LRYGB were made. This meta-analysis was conducted under the guidelines of preferred reporting items for systematic reviews and meta-analyses (PRISMA) 2009 [17].

### 2.2. Data Extraction

The full-texts of all included studies were independently reviewed by two authors (Ye and Xu) and data was extracted separately as well. In case of discrepancies, a third author (Min) was asked to discuss together until a consensus was achieved. The extracted data included study characteristics (author, publication year, study region, sample size, and procedures adopted), baseline patient demographics (age, gender, BMI, body weight, WC, and T2DM), and surgical outcomes, such as %EWL, changes in BMI (ΔBMI), postoperative BMI, weight loss, perioperative morbidity and mortality, and remission of T2DM. If rates and sample size were provided, number of events was calculated accordingly. Corresponding authors of included studies were contacted if needed.

### 2.3. Statistical Analysis

The statistics were performed using the software Review Manager (RevMan) version 5.2 (http://tech.cochrane.org/revman). The heterogeneity was calculated by Cochran's *χ* ^2^ and the *I* ^2^ test. According to the heterogeneity and the varying risk profiles of patients undergoing surgeries treated in different centers as well as the different indications for each surgical technique, the random effect model was first adopted to calculate the weighted mean difference (WMD) (continuous data) or risk ratio (dichotomous data) and their 95% confidence interval (95% CI). In case of no significant heterogeneities were seen among the included studies (*P* > 0.1, *I* ^2^ < 50%), the fixed effect model was used. The publication bias was evaluated by the funnel plot. For all analyses, *P* value less than 0.05 was considered statistically significant.

## 3. Results and Discussion

### 3.1. Literature Searching and Study Selection

**Figure 1:**
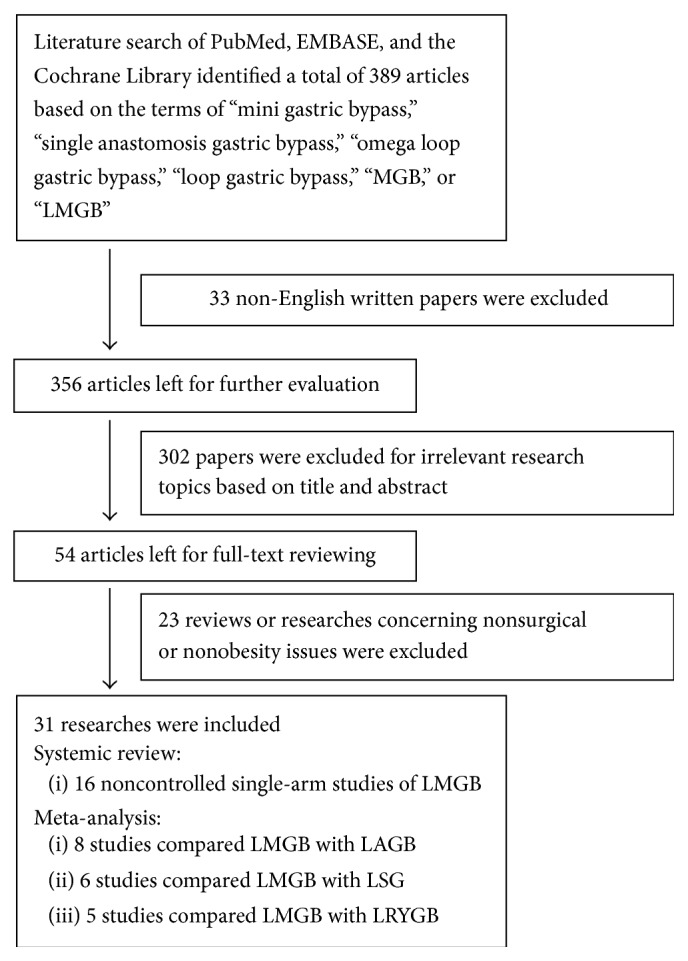
Flowchart of literature searching and study selection.

The flowchart of literature searching and study selection was shown in Figure 1. Retrieving of the databases identified a total of 389 literatures. Among them, 33 articles published in languages other than English were first excluded; another 302 papers were subsequently removed for irrelevant study topics after reviewing the titles and abstracts. Of the left 54 articles, full-texts were looked up and 23 studies which were reviews or reported nonsurgical, non-T2DM, or nonobesity related issues were excluded. The remaining 31 articles were included in this systematic review and meta-analysis: 16 noncontrolled single-arm case studies [15, 18–32] reported the surgical outcomes of LMGB (Table 1) and were used in the systematic review; among them, the participants of four studies [15, 21, 24, 30] were part of or overlapped with those of other three studies [19, 23, 29] and were not listed in Table 1. Of the left 15 studies, 8 studies [33–40] compared LMGB with LAGB, 6 studies [37, 38, 40–43] compared LMGB with LSG, and 5 studies [40, 44–47] compared LMGB with LRYGB; they were included in the meta-analyses, respectively. Two studies [37, 38] reported the results of LMGB versus LAGB and LSG, while one study [40] compared LMGB with LAGB, LSG, and LRYGB; they were used in different meta-analyses repeatedly.

### 3.2. Systematic Review of Noncontrolled Single-Arm LMGB Studies

Overall, 16 studies reported the outcomes of LMGB for obese patients. Study characteristics, baseline patients demographics, and 1-year postoperative results were shown in Table 1. The studies were mainly from western countries and regions, with participants ranging from 10 to 2410. Female patients were predominant and mean BMI was over 35 in most studies. The percentages of patients with T2DM varied from 0 to 100%.

In all studies, LMGB could be done successfully and few needed conversion to open surgery (Table 2). Surgical procedures of LMGB seemed not complicated since the learning curve soon reached a plateau and the OT was not long, ranging from 36.9 to 129 min. Generally, the OT decreased as the cases of LMGB increased. Rutledge [15] reported the first consecutive 1274 LMGBs in 2001 with a mean OT of 36.9 min. The OT reported by them was the shortest till now and subsequent studies with smaller sample size reported longer OT: Kular reported 1054 cases with mean OT of 52 min [31]; Wang, Noun, and Musella et al. reported mean OT around 90 min with case numbers of 423, 923, and 974, respectively [20, 28, 32]. In studies with smaller sample size, mean OTs exceeded 100 min. This might be attributed to the learning curve effect since for surgeons who continuously performed this surgery the OT longer than 150 min occurred mainly in the first 30 cases [20] and decreased to stable 50 min in the middle and late period [27]. Also, LMGB could be done with extremely low open surgery conversion rate. Carbajo et al. reported two conversions due to uncontrollable intra-abdominal hemorrhage [18]; four patients (4/2410) were converted to open surgery in the study by Rutledge and Walsh [19]; Kim and Hur reported one conversion for postoperative adhesion caused by previous nephrectomy [29]; in the study by Musella et al., 12/974 patients were converted while 8 of them had abdominal adhesions [32]. The rest of the studies conducted LMGB without conversion.

The main early perioperative morbidities were bleeding, leakage, and wound infection (Table 2). Carbajo et al. recorded 2 bleeding events within 24 hours after surgery and minilaparotomy was used for hemostasis [18]; Wang et al. noted 7 anastomosis bleeding cases, of which 5 were treated with proton pump inhibitors (PPIs) and transfusion while 2 needed reoperation [20]; Noun et al. reported 15 bleeding cases, of which 12 might be staple-line related bleeding [28]; Musella et al. reported 25 (2.5%) abdominal bleeding cases [32]. Reoperation was employed in case bleeding and leakage could not be resolved by conservative methods [18–20, 29]. Other complications like hernia, gastric stasis, and acute stenosis were few: Rutledge and Walsh reported 2 wound hernia cases (0.08%) [19]; Wang et al. reported one gastric stasis which was resolved by total parenteral nutrition for 2 weeks [20] and one efferent stasis in the study by Kim and Hur was also managed conservatively [29]; one anastomotic stenosis requiring endoscopic dilatation was reported by Chakhtoura et al. [22]. The mortalities were low and most were not surgery related: Carbajo et al. reported 2/209 deaths, pulmonary embolism in one and nosocomial pneumonia in another [18]; Rutledge and Walsh reported one death from myocardial infarction and another death from a perforated colon (2/2410) [19]; in the study by Wang et al., one patient died of leakage with sepsis and one died of chronic cerebral hypoxia [20]; one patient with BMI > 45 died in the study by Lee et al. [23] and one death was from Piazza et al. [27]; two deaths from myocardial infarction and myxedema caused epilepticus were recorded by Kular et al. [31]; Musella et al. reported one death from surgery related complication and one from pulmonary embolism [32]. The late complications including reflux, marginal ulcer, and iron deficiency anemia were also comparable with those reported by other bariatric surgeries and could be treated conservatively. Notably, iron deficiency anemia appeared more common: Rutledge et al. reported 4.9% anemia while Carbajo et al. reported 8.1% [18]; two recent studies by Kular et al. and Musella et al. also recorded high anemia incidences of 7.6% and 5.3% [31, 32]. In the study by Wang et al., 41 patients developed anemia, accounting for 28% of the patients visits during follow-up [20]. Luckily, nearly all cases could be managed with intravenous or oral iron supplements without surgical intervention.

The postoperative results of LMGB were recorded at different time points, ranging from 3 months to 6 years, and different parameters of surgical outcomes were used, including %EWL, ΔBMI, weight loss, postoperative BMI and weight, change in glucose, HbA1c (glycosylated hemoglobin), and remission of T2DM. LMGB was efficient in reducing body weight and improving T2DM. Nearly all patients achieved %EWL higher than 60% at one year after surgery except that Peraglie et al. reported 57% excess weight loss in patients with BMI > 60; however, the %EWL of these patients reached 65% two years after surgery. Regarding the long-term weight control effects, LMGB was also effective: Carbajo et al. and Piazza et al. reported 80% EWL at 18 months and 2 years, respectively [18, 27]; Kular et al. reported 91% and 85% at 2 and 5 years separately [31] and Musella et al. also achieved 77% %EWL five years after surgery [32]. Besides, LMGB significantly improved T2DM: of the nine studies which reported T2DM status, 7 studies achieved T2DM remission or improvement rate more than 80% and, notably, Wang et al. reported 100% 2-year remission with all the 79 T2DM patients who ceased medication [20]. In the study conducted by Kim and Hur [29], the participants of which were all diabetic patients, remission of T2DM was achieved in 53% patients in the first year and increased to 63% and 90% in the second and third year, respectively. Moreover, Musella et al. reported 84.4% T2DM remission rate five years postoperatively [32].

### 3.3. Meta-Analysis of LMGB versus LAGB, LSG, and LRYGB

In general, 15 studies were chosen for meta-analyses; the selection of studies for each meta-analysis was presented below. Characteristics of included studies were shown in Table 3. Due to the heterogeneous result reporting among studies, we extracted as much information as possible to make different comparisons in each analysis.

### 3.4. LMGB versus LAGB

**Figure 2:**
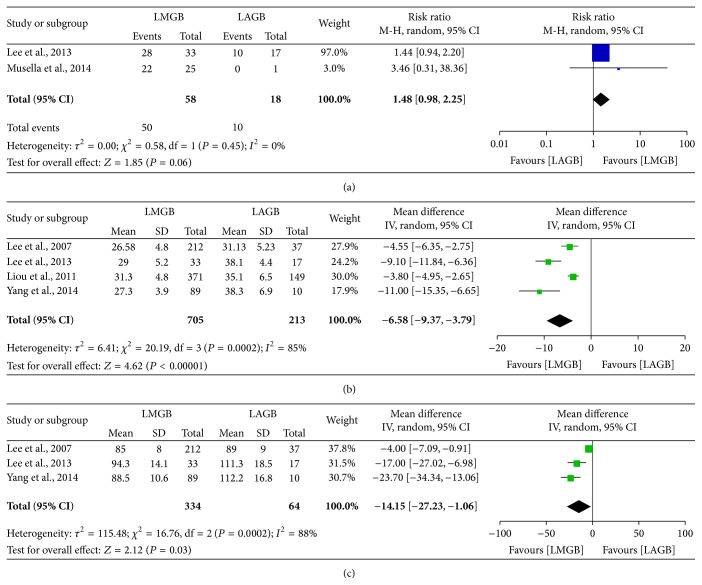
Meta-analysis comparing LMGB with LAGB. (a) Remission rate of T2DM. (b) Postoperative BMI. (c) Postoperative waist circumference. The estimates of the weighted risk ratio/mean difference in each study corresponded to the middle of each square and the horizontal line gave the 95% CI. The summary risk ratio/mean difference was represented by the middle of the solid diamond.

Overall, eight studies [33–40] compared the outcomes between LMGB and LAGB; two studies were removed for irrelevant research topics [33, 35] and one study [39] was excluded for data overlapping with another study with larger sample size [36]. According to the data extracted from the left five studies, we were only able to compare the overall remission rate of T2DM, postoperative BMI, and postoperative WC. The meta-analysis results were presented in Figure 2. Though LMGB showed a marginally higher T2DM remission rate than LAGB, only two studies included made this comparison less credible. Similarly, LMGB achieved lower BMI and reduced WC than LAGB; however, preoperative demographics varied among studies and in that case changes in BMI and WC would be more accurate in measuring weight loss than postoperative BMI and WC.

### 3.5. LMGB versus LSG

**Figure 3:**
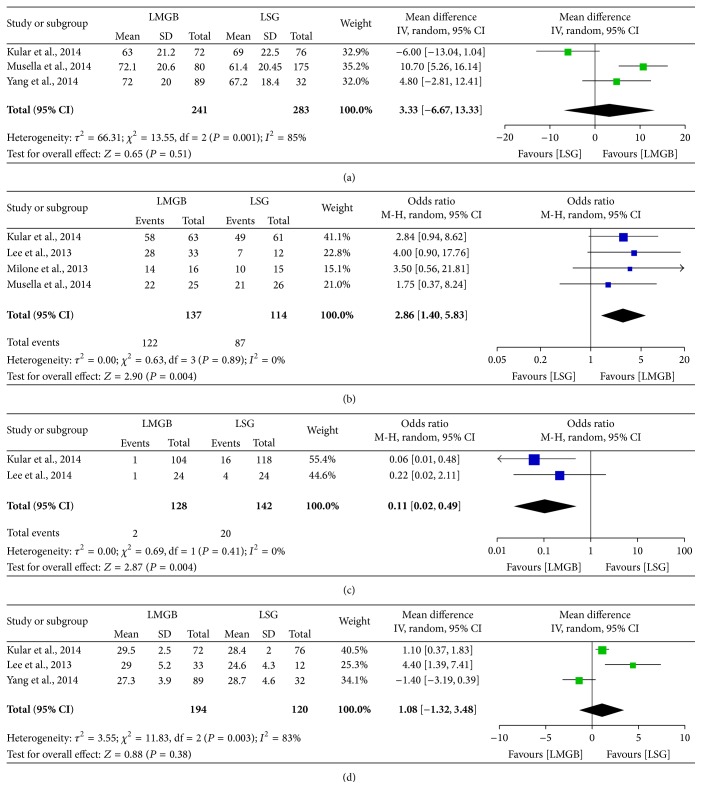
Meta-analysis comparing LMGB with LSG. (a) 1-year postoperative %EWL. (b) Overall remission rate of T2DM. (c) Revision surgery rate. (d) 1-year postoperative BMI.

Totally, six studies [37, 38, 40–43] reported either short- or long-term outcomes of LMGB and LSG. Remission of T2DM was better in patients receiving LMGB: meta-analysis of four studies [37, 38, 41, 42] showed higher remission rate of LMGB (89% versus 76%, *P* = 0.004) and no heterogeneity was observed (*I* ^2^ = 0%) (Figure 3). Specially, Kular et al. [42] reported the five-year T2DM remission rate and, still, LMGB was superior to LSG (92% versus 81%, *P* < 0.05). Notably, LMGB seemed to have lower revision rate than LSG (1.6% versus 14.1%, *P* = 0.004). Other parameters such as 1-year %EWL and 1-year postoperative BMI did not show significant differences between the two surgical approaches.

### 3.6. LMGB versus LRYGB

**Figure 4:**
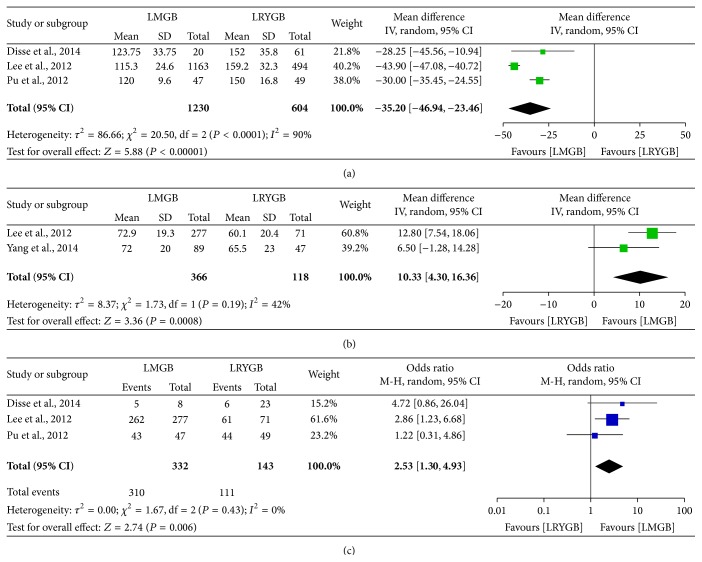
Meta-analysis comparing LMGB with LRYGB. (a) Operation time. (b) 1-year postoperative %EWL. (c) Overall remission rate of T2DM.

Five studies compared the outcomes of LMGB and LRYGB [40, 44–47] and one study [44] was excluded for data overlapping with that of a later one [45]. Compared with LRYGB, LMGB had significant shorter OT (WMD, −35.2, 95% CI, −46.94, −23.46; *P* < 0.00001). In terms of therapeutic effects, LMGB seemed more effective in weight loss (%EWL, *P* = 0.0008) and remission of T2DM (93.4% versus 77.6%, *P* = 0.006) than LRYGB (Figure 4).

## 4. Discussion

This systematic review and meta-analysis comprehensively evaluated the safety and effectiveness of LMGB and compared it with LAGB, LSG, and LRYGB. LMGB seemed efficient in reducing weight and improving T2DM with relatively low morbidity and mortality.

Proponents of LMGB believed that one less anastomosis than LRYGB made it much easier to learn and perform. Indeed, LMGB could be quickly learned: the learning curve for LMGB was 30 cases less than that for LRYGB [44] and it was estimated that 50 cases were needed to reach a stable OT. Specially, Wang et al. found that the operations which took longer than 150 min were mainly the first 30 cases and the OT curve decreased to a plateau after 50 cases [20]. Supporting this, Rutledge and Walsh and Piazza et al. reported that the OT stabilized at 30.3 and 50 min, respectively, in the later stage of their studies, shorter than the mean time of the whole study period [19, 27]. The simplification in surgical process of LMGB might causally decrease the OT. Additionally, in studies which reported the OTs of LMGB and other bariatric surgeries, LMGB always needed less time to be done [42, 44, 45, 47]. The simplified surgical technique also resulted in less blood loss [45], shorter hospitalization [42, 44], and faster bowel recovery [45]. However, these perioperative results were not systematically evaluated in previous studies and whether these benefits should be attributed to surgeons' experience or to the innate “safe” nature of LMGB should be further assessed.

Despite shorter OT, low perioperative morbidity, mortality, and fewer late complications of LMGB were noted. The highest overall complication rate was 9% among all enrolled studies [22] and, in studies with large cohort, it decreased to 5%, much lower than the overall complication rate (17%) of bariatric surgeries recently reported [48]. Also, studies with LMGB surgeries, more than 1000 cases, reported mortality of 0.2%, lower than the average 0.31% death rate of bariatric surgeries [48] and 0.5% of LRYGB [49]. Notably, anastomosis leakage and bleeding were the most frequent early complications of LMGB. Though one less anastomosis than LRYGB would surely reduce the chance of anastomotic leakage and bleeding, the long staple line on gastric pouch and remnant stomach might in turn increase such possibility [16]. Lee et al. [45] reported 0.2% major bleeding rate in the patients undergoing LMGB while reporting 1% in LRYGB (*P* < 0.05) and, in contrast with the early complication rate of 11.8% in LSG, LMGB achieved a much lower rate of 4.8% [42]. Since direct data comparing the bleeding and leakage rates of LMGB with those of other bariatric surgeries has not been reported, these results only partially indicated the noninferiority of LMGB to other bariatric procedures concerning morbidity and mortality.

The late complications including bile reflux, marginal ulcer, and iron deficiency anemia should be noted. Chronic alkaline reflux was associated with postoperative esophagitis and gastritis and would further empower carcinogenesis to the remnant stomach. However, remnant gastric cancer caused by bile reflux was rarely reported and reconstruction with Roux-en-Y gastric bypass proved to be quite safe [50–53]. In the reviewed studies, this complication was seldom reported and no patients required secondary surgery to relieve their symptoms [29, 32]; additionally, no dysplasia of any grade or remnant gastric cancers had been observed in studies reviewed. Still, one should recognize that similarity of LMGB to LRYGB does not ensure that the former has similar outcome concerning bile reflux since the effect caused by absence of a Roux limb is largely unknown to date [54]. Marginal ulcer of the remnant gastric pouch was another problem. The incidences of marginal ulcer of LMGB (0.6%–1.7%) were similar to or even lower than those of LRGYB (1.3%–4%) [55–57] and most cases could be treated effectively by PPI [57] and prophylactic PPI therapy postoperatively could inhibit development of marginal ulcer [56]. Iron deficiency anemia was frequent after LMGB; its incidence was around 5% and could be as high as 9.7%. Lee et al. compared LMGB with LRYGB and found that the former had lower hemoglobin levels 1 year after surgery and such low level persisted until 5 years postoperatively [44, 45]. The duodenal bypass might be one possible cause since it made part of the small bowel empty and thus became ineffective in food digestion and absorption; in addition, vitamin B12/folate shortage caused by deficiency of intrinsic factor was another potential reason. Fortunately, this complication could be treated with oral/injectable iron supplementation with few cases that needed transfusion or revisional surgery.

The surgical effects of LMGB seemed significant and durable. In studies which reported the 1-year postoperative %EWL, significant weight loss (%EWL > 50) was obtained in all and the %EWL continued to increase during the follow-up and was stable at 18 months, 2 years, and 5 years [29]. Kular et al. found that the 1-year %EWL of LMGB was comparable with LSG while, five years after surgery, %EWL of LMGB was significantly higher than that of LSG [42]. Lee et al. also reported higher 5-year %EWL for LMGB versus LRYGB and LMGB was more effective in reducing BMI than LAGB during the whole follow-up period [36, 39]. The results reported were quite heterogeneous among enrolled studies and meta-analyses were performed for different parameters in the three groups: LMGB achieved lower postoperative BMI and WC than LAGB, comparable 1-year BMI with LSG, and lower 1-year %EWL than LRYGB. LMGB also showed effects in T2DM remission. It was suggested that patients with extensive weight loss were more likely to achieve T2DM remission after bariatric surgery [58] and diversionary procedure like LMGB was more efficient in reducing weight [59]. In the review, 1-year remission rates higher than 80% [19, 20, 23, 32] and even 90% [27, 31] were noted. Significantly, Wang et al. reported that all the seventy-nine hyperglycemia patients resolved within 6 months and ceased medication thereafter [20]. The T2DM remission effect of LMGB seemed long-lasting since the HbA1c continued to decrease 3 years after surgery though BMI had gone to a plateau [29]. Inconformity in definition of T2DM remission made the meta-analyses only include limited studies, making the result less credible. Though the weight control and metabolic effects of bariatric surgeries had been well established [60–62], it remained controversial which technique should be given priority for obese patients. Compared to LAGB and LSG, LRYGB and LMGB were more invasive procedurally and extensive in restricting calorie intake. Most studies indicated pronounced weight loss effect of LSG and LRYGB compared to LAGB while the latter seemed to be less invasive and much safer [63]. Similarly, LRYGB was more effective for the surgical treatment of T2DM and control of metabolic syndrome whereas LSG proved to be safer and had few complications [64, 65]. In contrast, comparable low morbidity and mortality and similar T2DM remission rates between LRYGB and LSG had also been recorded [66] and prospective study with 5-year follow-up found similar results of LRYGB and LSG [67]. Additionally, for patients who failed sleeve gastrectomy, conversion to LMGB was found to be feasible, safe, and effective [68]. Thus, further trials to comprehensively evaluate the treatment choices of bariatric procedures are needed.

There existed some limitations in this systematic review and meta-analysis. First, most studies were performed in single-arm design without controlled groups and the follow-ups of included studies were not long enough, making the late complications and surgical effects underestimated. Second, inconformity in result reporting among the studies made it difficult to collect enough information to compare LMGB with other bariatric procedures. Under such conditions, it was difficult to draw a solid conclusion on the utility of LMGB. Third, the definition on T2DM remission used in different studies varied and recent study had found that the remission rate could change dramatically depending on the criteria used [69]. Thus direct comparison of T2DM remission rate between different studies was less accurate and the pooled size effect might not reflect the surgical effects.

## 5. Conclusions

Compared with LAGB, LSG, and LRYGB, LMGB was at the least not inferior in weight loss and T2DM remission and, at the same time, it had few complications. Nevertheless, there still remain some issues to be further clarified, that is, the long-term (>5 years) effects on weight loss and T2DM remission, late complications, and their treatment. To this end, future clinical trials with prospective design are still needed to demonstrate its utility and establish the guidelines for LMGB.

## Figures and Tables

**Table 1:** General characteristics of the noncontrolled single-arm LMGB studies.

Study characteristics			Baseline patient demographics						1-year postoperative results			
Author	Year	Region	Number (M/F)	Age (range)	BMI (kg/m^2^)	Weight (kg)	WC (cm)	T2DM patients	%EWL	BMI (kg/m^2^)/ΔBMI	Weight/weight loss (kg)	Remission or improvement of T2DM
Carbajo et al. [18]	2005	Spain	209 (37/172)	41 (14–66)	48 (39–86)	NR	NR	NR	75%, 80% at 18 months	NR/NR	NR/NR	NR

Rutledge and Walsh [19]	2005	USA	2410 (361/2049)	39 (14–78)	46 ± 7 (34–74)	NR	NR	24%	80%	29/NR	NR/59	83%

Wang et al. [20]	2005	Taiwan	423 (87/336)	30.6 ± 9.3 (19–62)	44.2 ± 7 (35.5–72.1)	120.3 ± 23.4 (89–212.7)	NR	79	69.3%	29.2/NR	NR	100%

Chakhtoura et al. [22]	2008	France	100 (23/77)	40.9 ± 11.5 (17.5–62.4)	46.9 ± 7.4 (32.8–72.4)	131 ± 23.1 (82–203)	NR	None	63 ± 14%	31.9 ± 5.7/NR	89.8 ± 18.4/NR	NR

Lee et al. [23]	2008	Korea	44 (6/38) 114 (33/81) 43 (19/24)	39 ± 8.9 34.8 ± 9.2 28.2 ± 8.7	31.7 ± 2.7 40 ± 2.8 51.4 ± 6	143.1 ± 9.8 107.6 ± 5.1 81.2 ± 5.1	103.4 ± 7.1 117.6 ± 14.7 137.8 ± 15.3	All	NR	NR/8.5 ± 2.2 NR/12.4 ± 3.2 NR/17.9 ± 5.8	91.4 ± 9.8/NR 79.2 ± 5.5/NR 60.4 ± 3.3/NR	87.1%

Peraglie [25]	2008	USA	16 (2/14)	40 (27–61)	62.4 (60–73)	166 (150–193)	NR	NR	57%, 65% at 2 years	NR	NR/63	NR

Kim and Hur^*∗*^ [26]	2011	Korea	10 (2/8)	46.9 (29–59)	27.2 (25.3–29.9)	NR	NR	All	NR	NR/23.4 (21.3–25.6)	NR/NR	70%

Piazza et al. [27]	2011	Italy	197 (50/147)	37.9 (20–55)	52.9	NR	NR	NR	65%, 80% at 2 years	39.4 ± 4.2/NR	NR/NR	90%

Noun et al. [28]	2012	Lebanon	923 (315/606)	32.77 ± 10 (13–70)	42.5 ± 6.39 (35–75)	121.64 ± 23.85 (80–240)	NR	19%	69.9% ± 23.1%	28.3 ± 4.8/NR	80.3 ± 14.2/NR	85%

Kim and Hur [29]	2014	Korea	107 (54/53)	46 ± 11 (21–74)	25.3 ± 3.2 (18–30)	NR	NR	All	NR	22.9 ± 3.0/NR	NR/NR	53%, 63% at 2 and 90% at 3 years

Kular et al. [31]	2014	India	1054 (342/712)	38.4 ± 9.6	43.2 ± 7.4	128.5 ± 25.2	118.2 ± 15.7	674 (64%)	85%, 91% at 2 and 85% at 5 years	26.2 ± 3.1/NR	NR	93.2% of remission; 98% of improvement

Musella et al. [32]	2014	Italy	974 (475/499)	39.4	48 ± 4.58	NR	NR	224 (22.9%)	70.12 ± 8.35%, 77% at 5 years	31.88 ± 4.91/NR	91.5 ± 18.5/NR	87%, 84.4% at 5 years

NR: not reported; ^*∗*^postoperative results at 6 months.

**Table 2:** Surgical characteristics and complications of the noncontrolled single-arm LMGB study.

Author	Number of surgeries	Time (min)	Conversion to open surgery	Revision rate	Blood loss (mL)	Reoperation	Hospital stay	Perioperative mortality	Overall complication rate	Wound infection	Leakage	Bleed	Reflux	Dyspepsia and/or ulcer	Iron deficiency anemia
Carbajo et al. [18]	209	93^*∗*^ (70–155)	2 (0.9%)	NR	NR	3 (1.3%)	1.5	2 (0.9%)	4.8%	NR	4 (1.9%)	2 (0.9%)	0	NR	17 (8.1%)
Rutledge and Walsh [19]	2410	37.5	0.17%	NR	25–50	NR	NR	0.08%	5.9%	0.12%	1.08%	NR	NR	5.6%	4.9%
Wang et al. [20]	423	95 ± 41.5 (40–310)	0	NR	NR	3 (0.7%)	5.0 ± 1.8 (3–15)	2 (0.47%)	6%	5	9 (2.1%)	7 (1.7%)	NR	34 (8.0%)	41
Chakhtoura et al. [22]	100	129 ± 37 (80–240)	0	NR	NR	3 (3%)	8.5 ± 2.2 (6–21)	0	9%	NR	0	2 (2%)	2 (2%)	1 (1%)	NR
Lee et al. [23]	201	116.3 ± 40.9	NR	NR	34.3 ± 33.2	NR	6.6 ± 5.8	1 (0.5%)	7 (3.5%)	NR	NR	NR	NR	NR	NR
Peraglie [25]	16	78 (41–147)	0	0	NR	0	1.2	0	0	NR	NR	NR	NR	NR	NR
Kim and Hur [26]	10	150.5 (100–190)	0	0	NR	0	5.3 (4–7)	0	0	0	0	0	0	0	NR
Piazza et al. [27]	197	120 (90–170)	0	0	NR	0	5	1 (0.5%)	4.1%	0	0	6	2	3	NR
Noun et al. [28]	923^*∗∗*^	94 ± 4.65 (80–175)	—	0	NR	0	1.85 ± 0.8	0	2.7%	NR	4 (0.42%)	15 (1.61%)	0	6 (0.65%)	NR
Kim and Hur [29]	172	87 ± 34 (45–210)	1	2	NR	1	4.5 ± 1 (3–7)	0	5 (2.9%)	NR	1	2	NR	22	12
Kular et al. [31]	1054	52 ± 18.5	0	NR	NR	2 (0.2%)	2.5 ± 1.3	2 (0.18%)	53 (5.9%)	4 (0.3%)	2 (0.1%)	3 (0.2%)	18 (2.0%)	5 (0.6%)	68 (7.6%)
Musella et al. [32]	974	95 ± 51.6	12 (1.23%)	7 (0.8%)	NR	20 (2%)	4 ± 1.7	2 (0.2%)	54 (5.5%)	NR	10 (1%)	25 (2.5%)	8 (0.9%)	14 (1.7%)	44 (5.3%)

NR: not reported; ^*∗*^patients of primary surgery; ^*∗∗*^data including open MGB.

**Table 3:** General characteristics of studies included in meta-analyses.

Study characteristics	Year	Surgeries (LMGB versus )	Number of patients	Gender (female/male)	Age (years)	BMI (kg/m^2^)	Remission of T2DM^#^	%EWL^#^
Lee et al. [34]	2007	LAGB	212 versus 37	177 versus 72	33 ± 9	26.58 ± 4.8 versus 31.13 ± 5.23	NR	78.54 ± 26.87 versus 43.65 ± 26.08^*∗*^

Liou et al. [36]	2011	LAGB	371 versus 149	266/105 versus 83/66	30.7 ± 8.3 versus 31.9 ± 9.2	42 ± 6.2 versus 41.9 ± 6.3	NR	NR

Lee et al. [37]	2013	LAGB LSG	33 versus 17 33 versus 12	39 versus 23	31.8 ± 9.2	41.7 ± 7.3 versus 41.7 ± 5 41.7 ± 7.3 versus 39.6 ± 0.7	84.8% versus s 58.8%^*∗*^ 84.8% versus 58.3%^*∗*^	NR

Musella et al. [38]	2014	LAGB LSG	80 versus 120 80 versus 175	38/42 versus 75/45 38/42 versus 123/52	34.8 versus 39.5 34.8 versus 38.2	50.8 versus 42.3 50.8 versus 47.9	— 88% versus 80.7%	79.5 versus 58.2^*∗*^ (36 months) 79.5 versus 68.3^*∗*^ (36 months)

Yang et al. [40]	2014	LAGB LSG LRYGB	89 versus 10 89 versus 32 89 versus 47	68/21 versus 5/5 68/21 versus 13/19 68/21 versus 35/12	32.1 ± 10.3 versus 34.8 ± 12.2 32.1 ± 10.3 versus 33.9 ± 9.4 32.1 ± 10.3 versus 33.2 ± 9.4	41.7 ± 5.6 versus 41.5 ± 6.8 41.7 ± 5.6 versus 42.4 ± 8.9 41.7 ± 5.6 versus 42.7 ± 7.0	NR	72 ± 20 versus 16.1 ± 14.3^*∗*^ 72 ± 20 versus 67.2 ± 18.4^*∗*^ 72 ± 20 versus 65.5 ± 23^*∗*^

Milone et al. [41]	2013	LSG	16 versus 15	8/8 versus 8/7	39.3 ± 2.3 versus 37.26 ± 3.7	45.8 ± 5 versus 43.6 ± 2.99	87.5% versus 66.7%	NR

Kular et al. [42]	2014	LSG	72 versus 76	NR	NR	44 ± 3.1 versus 42 ± 5.2	92% versus 81%^*∗*^	63 ± 21.2 versus 69 ± 22.5^*∗*^

Lee et al. [43]	2014	LSG	30 versus 30	22/8 versus 22/8	44.6 ± 8.6 versus 46.4 ± 8.1	30.2 ± 2.2 versus 31 ± 2.8	NR	NR

Lee et al. [45]	2012	LRYGB	1163 versus 494	850/313 versus 362/132	32.3 ± 9.1 versus 33.5 ± 9.3	41.1 ± 6.1 versus 40.5 ± 5.8	NR	72.9 ± 19.3 versus 60.1 ± 20.4^*∗*^

Pu et al. [46]	2012	LRYGB	47 versus 49	23/24 versus 21/28	44.44 ± 3.02 versus 44 ± 2.25	26.5 ± 2.11 versus 26.3 ± 2.19	91.4% versus 90.3%	NR

Disse et al. [47]	2014	LRYGB	20 versus 61	14/6 versus 43/18	49.5 versus 47	40.1 versus 42.3	62.5% versus 26%	89 versus 71^*∗*^

NR: not reported; ^*∗*^ *P* < 0.05; ^#^one-year postoperative results.
